# The evaluation of five serological assays in determining seroconversion to peste des petits ruminants virus in typical and atypical hosts

**DOI:** 10.1038/s41598-023-41630-3

**Published:** 2023-09-08

**Authors:** Matthew Tully, Carrie Batten, Martin Ashby, Mana Mahapatra, Krupali Parekh, Satya Parida, Felix Njeumi, Brian Willett, Arnaud Bataille, Genevieve Libeau, Olivier Kwiatek, Alexandre Caron, Francisco J. Berguido, Charles E. Lamien, Giovanni Cattoli, Gerald Misinzo, Julius Keyyu, Daniel Mdetele, Francis Gakuya, Sanne Charles Bodjo, Fatima Abdelazeem Taha, Husna Mohamed Elbashier, Abdelmalik Ibrahim Khalafalla, Abdinasir Y. Osman, Richard Kock

**Affiliations:** 1 The Pirbright Institute, Pirbright, United Kingdom; 2 Food and Agriculture Organization (FAO), United Nations, Rome, Italy; 3 MRC-University of Glasgow Centre for Virus Research (UoG), Glasgow, United Kingdom; 4 ASTRE, University of Montpellier, CIRAD, INRA, MUSE, Montpellier, France; 5 Animal Production and Health Laboratory, Joint FAO and IAEA Centre for Nuclear Applications in Food and Agriculture, Department of Nuclear Sciences and Applications, International Atomic Energy Agency, Friedenstrasse 1, 2444 Seibersdorf, Austria; 6 SACIDS Foundation for One Health, Sokoine University of Agriculture, Morogoro, Tanzania; 7 Tanzania Wildlife Research Institute (TAWIRI), Arusha, Tanzania; 8 Ministry of Livestock and Fisheries, Dodoma, Tanzania; 9 Wildlife Research & Training Institute (WRTI), Karagita, Kenya; 10 Pan African Veterinary Vaccine Centre for African Union (AU-PANVAC), Debre Zeit, Ethiopia; 11 Central Veterinary Research Laboratories (CVRL), Khartoum, Sudan; 12 Tumbool Camel Research Centre (TCRC), Tamboul, Sudan; 13 Abu Dhabi Agriculture and Food Safety Authority (ADAFSA), Abu Dhabi, United Arab Emirates; 14 Faculty of Veterinary Medicine, University of Khartoum, Khartoum, Sudan; 15 National Institute of Health (NIH), Ministry of Health, Mogadishu, Somalia; 16 Royal Veterinary College (RVC), London, United Kingdom

**Keywords:** ELISA, Immunological models, Molecular engineering, Virology, Biological techniques, Diseases

## Abstract

Peste des petits ruminants (PPR) is an infectious viral disease, primarily of small ruminants such as sheep and goats, but is also known to infect a wide range of wild and domestic Artiodactyls including African buffalo, gazelle, saiga and camels. The livestock-wildlife interface, where free-ranging animals can interact with captive flocks, is the subject of scrutiny as its role in the maintenance and spread of PPR virus (PPRV) is poorly understood. As seroconversion to PPRV indicates previous infection and/or vaccination, the availability of validated serological tools for use in both typical (sheep and goat) and atypical species is essential to support future disease surveillance and control strategies. The virus neutralisation test (VNT) and enzyme-linked immunosorbent assay (ELISA) have been validated using sera from typical host species. Still, the performance of these assays in detecting antibodies from atypical species remains unclear. We examined a large panel of sera (n = 793) from a range of species from multiple countries (sourced 2015–2022) using three tests: VNT, ID VET N-ELISA and AU-PANVAC H-ELISA. A sub-panel (n = 30) was also distributed to two laboratories and tested using the luciferase immunoprecipitation system (LIPS) and a pseudotyped virus neutralisation assay (PVNA). We demonstrate a 75.0–88.0% agreement of positive results for detecting PPRV antibodies in sera from typical species between the VNT and commercial ELISAs, however this decreased to 44.4–62.3% in sera from atypical species, with an inter-species variation. The LIPS and PVNA strongly correlate with the VNT and ELISAs for typical species but vary when testing sera from atypical species.

## Introduction

Peste des petits ruminants (PPR), also known as ovine rinderpest, is a contagious transboundary animal disease that primarily affects small ruminants such as goats and sheep^1,2^, considered typical hosts, and is widespread across Africa, the Middle East and Asia^2^. PPR is estimated to cause up to $2.1 billion a year in losses globally with the majority of this sum shouldered by small-scale rural farmers^3^. The causative *Morbillivirus*, peste des petits ruminants virus (PPRV) of the *Paramyxoviridae* family, is a negative sense, single-stranded RNA virus with a genome ~ 16 kb in length, categorized into genetic lineages I–IV. The haemagglutinin, fusion and nucleocapsid proteins, encoded by the H, F and N genes respectively, are the main antigenic components of the virion. These elicit a strong cell-mediated and humoral immune response during PPRV infection, with antibodies to H and F being protective and those to N being non-protective^4,5^. These antibodies form the primary targets for serological assays to detect seroconversion to PPRV.

With host morbidity and mortality rates as high as 80% in naïve populations, PPR presents a considerable risk to food security, agricultural practices, biodiversity and the livelihoods of those living in affected areas^6^. In addition to sheep and goats, the disease has also been reported in some captive and free-ranging wild and domestic Artiodactyls such as African buffalo (*Syncerus caffer*), camel (*Camelus *sp.) and antelope species such as impala (*Aepyceros melampus*)^7^. Bovids, camelids and Suidae species were generally considered to be dead-end hosts, playing little or no part in transmission, but recent studies have shown that these atypical hosts may develop clinical symptoms, spread disease and seroconvert in captive and experimental settings, impacting their epidemiological significance^8–11^. The disease presentation and seroconversion rates in these species are therefore still being investigated. A notable natural outbreak of PPR occurred between 2016 and 2017 when wild saiga antelope (*Saiga tatarica mongolica*) populations in Mongolia were severely impacted, with livestock animals highlighted as the likely source of PPRV transmission^12^.

In 2011 the Food and Agriculture Organization (FAO) and the World Organisation for Animal Health (WOAH) announced the eradication of rinderpest disease following a successful programme of diagnosis, vaccination and surveillance. Given the similarities in their spread, host range and clinical manifestation, PPR has been targeted by a comprehensive campaign with a view to ridding the world of this destructive disease by 2030^13^. Understanding the role of atypical hosts, both domestic and wild Artiodactyls, in the maintenance and spread of PPR in complex ecosystems is crucial to support the global PPR eradication programme. The standardisation of approaches to outbreak control, vaccine design and distribution and laboratory diagnosis, including serological techniques, are the core components of global eradication strategies^14^. Thus far, comprehensive data are not available regarding the current epidemiological role of atypical host species in complex ecosystems.

Serological testing (the measurement of serum antibody content) is recommended by WOAH as a method of detecting exposure to PPRV and/or vaccination and can support diagnosis^15^. For surveillance strategies, several cost-effective and widely used methods for PPR serology are available and these are generally preferable to molecular techniques due to the short periods of viraemia during infection. However, some of the existing tests have imperfect specificity^16^ and thus the need for multiple testing increases the chance of false positive results in healthy, non-exposed populations while poor sensitivity can lead to missed opportunities to detect disease. The virus neutralisation test (VNT) is generally accepted as the gold standard serological test for PPRV and, whilst sensitive, requires facilities capable of containing and handling live virus with appropriate biosecurity measures. Infrastructure of this calibre is expensive to install and maintain, making the test an impractical choice in many settings, especially in resource-constrained locations where the disease is endemic. The commercially available competition enzyme-linked immunosorbent assay (cELISA) from ID VET, which detects antibodies raised against PPRV nucleocapsid (N) protein, offers a practical and effective solution to the biosafety limitations of the VNT. However, as both of these tests have only been validated for use in sheep and goats (and in the case of the ID VET ELISA, pigs and camels) they may not demonstrate immunity in cases beyond typical hosts as N-antibodies are non-protective^17^. Alternatively, AU-PANVAC produce a monoclonal antibody (mAb)-based blocking ELISA targeting antibodies raised against PPRV haemagglutinin (H) which are neutralising. This method has also undergone validation using goat and sheep sera. Data from the manufacturer suggests this assay has a diagnostic specificity and diagnostic sensitivity of 100% and 93.74%, respectively^18^.

The Animal Production and Health Laboratory of the Joint FAO and IAEA Centre developed a luciferase immunoprecipitation system (LIPS) for the rapid detection of anti-PPRV Nucleocapsid (N) antibodies in serum samples^1^. This PPR-LIPS is highly sensitive and specific to PPR as the target protein can be customised using known genetic sequences. In parallel, the University of Glasgow Centre for Virus Research has developed a pseudotyped virus neutralisation assay (PVNA) for morbilliviruses including PPRV, another assay for which targets can be customised on a genetic level. The PVNA test enables distinctions to be made between PPRV-specific neutralisation and cross-neutralising responses elicited by infection with other species of morbillivirus^19^.

In order to examine and compare the sensitivity and specificity of these serological assays, in both typical domestic sheep and goats and atypical domestic and wildlife species, a large panel of diverse sera, obtained from field sampling, has been tested and the agreement between methods calculated. We aim to elucidate the performance of each assay beyond that of typical host species to bring greater confidence to serological surveillance.

## Materials and methods

### Serological panel

A total of 793 serum samples were used in this study; 91 sera of livestock sheep and goats (herein referred to as typical) from pastoral herds and 702 sera from free-ranging wildlife and other domestic species (herein referred to as atypical), in their natural habitats, collected in the period 2015–2022 (Table 1). These sera were examined for antibody content using the VNT, ID VET ELISA and AU-PANVAC ELISA. From this panel, a smaller panel of 30 sera was selected, including samples with high, low and undetectable antibody titres, and distributed to the Animal Production and Health Laboratories of the Joint FAO/IAEA Centre for Nuclear Techniques in Food and Agriculture in Austria and the University of Glasgow (UoG) for testing using the LIPS and PVNA respectively. All samples originated from countries where PPR is endemic, spanning Tanzania, Kenya, Sudan, Mongolia and Pakistan. Sera used in this study were held by The Pirbright Institute, collected from previous studies:^6,7,10,12,20^. Sera were heat inactivated, at 56 °C for 2 h, prior to testing.

**Table 1:** Sample sera from livestock and wildlife.

Species type	Host species	Total number of samples (n)
Typical	Sheep	32
	Goat	48
	Labelled ‘Sheep and Goat’	11
Atypical	Dromedary	105
	African buffalo	262
	Cattle	41
	Wildebeest	16
	Waterbuck	7
	Topi	11
	Impala	31
	Hartebeest	2
	Lesser Kudu	1
	Gerenuk	1
	Grant’s Gazelle	147
	Thomson’s Gazelle	12
	Warthog	34
	Saiga	24
	Yak	1
	Alpaca	3
	Llama	2
	Pig	2
	Total	793

### Virus neutralisation test (VNT)

The PPR virus neutralization test (VNT) was performed using a recombinant Nigeria 75/1 PPRV strain (rPPRV/eGFP Nig 75/1) expressing green fluorescent protein (GFP)^21^ according to the recommendations of the WOAH terrestrial manual^15^. Sera were diluted 1/10 or 1/2 and subsequently serially diluted, two-fold, with 100 μl/well in Dulbecco's Modified Eagle Medium (DMEM), supplemented with 1% penicillin/streptomycin, in a 96-well flat bottom tissue culture plate.

Sera diluted in DMEM were incubated for 1 h at 37 °C with 100 TCID_50_ rPPRV/eGFP Nig 75/1. After incubation, 10^4^ Vero cells stably transfected to express dog SLAM (VDS) cells were added per well and the plates were incubated at 37°C in the presence of 5% CO_2_ for 7 days.

The cells were examined under 461/488 nm light (Olympus CKX53 with CoolLED pE-300) for the presence of GFP fluorescence. Each well, where GFP was observed, was recorded as ‘positive’ and a neutralising antibody titre was derived from the dilution at which half of the wells showed full neutralisation, i.e. no GFP. The neutralising antibody (nAb) titre was expressed as the serum fraction at which 50% neutralisation was observed. Titres of 1/10 or greater were considered positive. For the purposes of this study, where a nAb titre of < 1/10 was observed, but above the limit of detection for the test, an inconclusive result was assigned.

### ID VET PPRV cELISA

Serum samples were tested for the presence of anti- nucleoprotein (NP) antibodies in the ID VET PPRV ELISA following the manufacturer’s instructions^17,22^.

In brief, wells of a 96-well plate were coated with purified recombinant PPRV nucleoprotein (NP) and diluted sera were added to the wells and incubated at 37 °C for 45 min. Following a wash step, anti-NP-peroxidase (HRP) conjugate was added and incubated at 21 °C for 30 min. This formed a complex with the remaining free NP epitopes. Plates were washed again to remove excess conjugate and the substrate solution, 3,3′,5,5′-Tetramethylbenzidine (TMB), was then added and the plate incubated at 21 °C for 15 min in the dark. A ‘stop’ solution of 0.5 M H_2_SO_4_ was then added and the optical densities (OD) of the wells were read at 450 nm in an absorbance plate reader (Multiskan FC Microplate Photometer with SkanIT 6.1 software). The competition percentage (S/N%) for each was calculated using the following formula:

$${\text{S}}/{\text{N}}\% = \left( {{\text{OD}}\;{\text{sample}}/{\text{OD}}\;{\text{negative}}\;{\text{control}}} \right) \times {1}00$$

Classification of positive/negative/doubtful were as per the manufacturer’s instructions, summarised in supplementary table S1.

### AU-PANVAC PPRV bELISA

Serum samples were tested for the presence of anti-H antibodies in the AU-PANVAC PPRV ELISA, following the manufacturer’s instructions^18,23^.

In brief, wells of a 96-well plate were coated with crude, inactivated PPRV antigen (Nig 75/1 from Vero culture lysate). Sera, diluted in PBS-T containing 5% skimmed milk, were added to the wells and incubated at 18–25 °C for 1 h. After a wash step, the anti-H mAb conjugate, C4F3-HRP (diluted in PBS-T-milk) was added to bind free epitopes and the plate incubated at 18–25 °C for 45 min. Another wash step was performed to remove excess conjugate, then TMB substrate was added and the plate incubated at 37 °C for 15 min in the dark. Following incubation, 1 M H_2_SO_4_ was added to stop all reactions and the OD of the wells were read at 450 nm in an absorbance plate reader (Multiskan FC Microplate Photometer with SkanIT 6.1 software). The percentage inhibition (PI) for each was calculated using the following formula:

$${\text{PI}}\left( \% \right) = {1}00{-}\left( {\left( {{\text{OD}}\;{\text{sample}}{-}{\text{OD}}\;{\text{control}}\;{\text{buffer}}} \right)/\left( {{\text{OC}}\;{\text{negative}}\;{\text{control}}{-}{\text{OD}}\;{\text{control}}\;{\text{buffer}}} \right)} \right) \times {1}00$$

Classification of positive/negative/doubtful were as per the manufacturer’s instructions, summarised in supplementary table S1.

### Luciferase immunoprecipitation system (LIPS)

The protocol for LIPS has been previously described^1,24^. Briefly, total luciferase activity was determined by mixing 1 μl of crude fusion protein extract and 9 µl of phosphate buffered saline (PBS) to 100 μl of coelenterazine substrate (Promega) in a white 96 well-plate (Sterilin). The emission of relative light units (RLU) was detected by a luminometer (Berthold Centro LB 960, Berthold Technologies, Bad Wildbad Germany) with a cumulative read of 5 s. Based on the RLU measured, the volume of fusion protein extract required to produce 1 × 10^7^ RLU was determined.

LIPS reactions were carried out by mixing 40 μl of buffer A (50 mM Tris, pH 7.5, 100 mM NaCl, 5 mM MgCl_2_, 1% Triton X-100, 250 mM Glycine), 10 μl of diluted serum (diluted 1/10 in buffer A) and 50 μl of buffer A containing enough fusion protein extract to generate 1 × 10^7^ RLU (as calculated above) in each well of a 96-well-plate. The mixture was incubated at room temperature for 1 h with gentle shaking. The mixture was then transferred to a 96 well Multi-Screen HTS filter plate (Millipore) and incubated with 5 μl of Ultralink immobilized protein A/G beads (Pierce Biotechnology Inc) for 1 h at room temperature with gentle shaking, and then washed Eight times with buffer A and twice with PBS using a vacuum manifold. 50 μl of coelenterazine substrate was added to each well and the light emission was read for 5 s using a luminometer.

Results were given as an average of two replicates per run (usually two runs) minus the blank. Threshold limits were calculated as a mean of the negative values plus 3 or 5 standard deviations (STD). Values below the mean plus 3 STD were considered negative, values above the mean plus 5 STD were positive and values in between the two were considered borderline (see supplementary data, table S1).

### Pseudotyped virus neutralisation assay (PVNA)

HEK293 and HEK293T cells were maintained in DMEM supplemented with 10% foetal bovine serum, 100 IU/ml penicillin, 100 μg/ml streptomycin, 2 mM glutamine and 0.11 mg/ml sodium pyruvate (complete medium). Media for 293T cells and 293 cells stably expressing canine SLAM were supplemented with 400 µg/ml G418 (Geneticin®, Life Technologies Ltd.). All media and supplements were obtained from Life Technologies Ltd., Paisley, UK.

Recombinant vesicular stomatitis virus (VSV) in which the glycoprotein (G) gene has been deleted (VSVΔG) and replaced with firefly luciferase (luc) has been described^25,26^ and was kindly provided by Michael Whitt, Memphis, TN, USA. An initial stock of VSVΔGluc bearing VSVG was used to infect 293T cells transfected with the VSV-G expression vector pMDG^27^. VSVΔGluc (VSVG) pseudotypes were recovered, titrated on 293T cells and used to prepare a working stock of VSVΔGluc (VSV-G) pseudotypes. The construction of the expression plasmids for the H and F genes of the vaccine strain of PPRV (PPRV/Nigeria/75/1) has been previously described^28^.

To prepare VSVΔGluc pseudotypes, 293T cells were transfected with the H and F expression vectors from PPRV, followed by super-infection with VSVΔGluc (VSVG) as previously described^25,26^. Supernatants were harvested 48 h post-infection, aliquoted and frozen at − 80 °C. The titre of each viral pseudotype stock was estimated by preparing serial dilutions in triplicate and plating onto 293dogSLAM cells followed by incubation for 48–72 h at 37 °C, at which time luciferase substrate was added (Steadylite plus™, Perkin Elmer) and the signal analysed on an Ensight multimode plate reader (Perkin Elmer). The viral titre [50% tissue culture infectious dose (TCID_50_)] was calculated using the Spearman–Kärber formula^29^.

A quantity of 2 × 10^4^ 293-dogSLAM cells were plated into each well of a white 96-well flat-bottomed cell culture plate (Culturplate-96, Perkin Elmer, Coventry, UK). Four-fold serum dilutions were prepared in triplicate in complete medium ranging from 1:32 to 1:65,536. The diluted serum samples were then added to the 293-dogSLAM cells followed by 2.5 × 10^3^ TCID_50_ of VSVΔG(CDV) pseudotype. Plates were incubated for 48–72 h at 37 °C, at which time luciferase substrate was added (Steadylite plus™, Perkin Elmer) and the signal analysed on an Ensight multimode plate reader (Perkin Elmer). Antibody titres were calculated by interpolating the point at which there was a 90% reduction in luciferase activity (90% neutralisation, inhibitory concentration 90 or IC_90_) (see supplementary data, table S1).

### Ethical statement

All samples used in this study were procured from previous ethically approved studies, referenced in this manuscript, and were held at the Pirbright Institute prior to testing. All ethical standards of the journal are adhered to. No further ethical approval is therefore stated.

## Results

**Figure 1:**
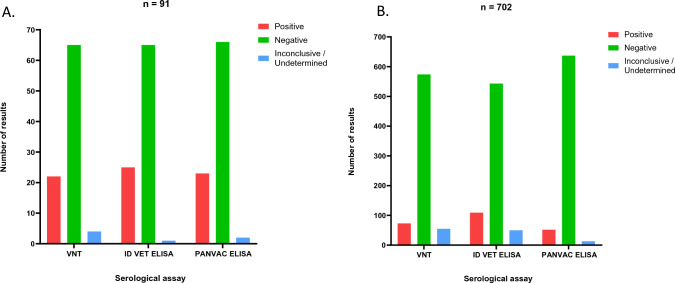
The number of positive, negative and inconclusive/undetermined results from the large sera panel (n = 793) when tested using the VNT, ID VET ELISA and AU-PANVAC ELISA. Graph (**A**) depicts typical hosts (sheep and goat) and graph (**B**) depicts atypical hosts.

The 91 sera from typical livestock, comprising sheep and goat, were tested in the VNT, ID VET ELISA and AU-PANVAC ELISA. The VNT detected 22 positives, 65 negatives and four were considered inconclusive due to the observation of a neutralising antibody titre of < 1/10 (but still above the limit of detection) or because of bacterial contamination obscuring the results (Fig. 1A). The ID VET ELISA detected 25 positives, 65 negatives and 1 inconclusive and the AU-PANVAC ELISA detected 23 positives, 66 negatives and 2 inconclusive results (Fig. 1A). Of the positive results generated, three were unique to the ID VET kit and two were unique to the AU-PANVAC kit. There were no unique positives identified using the VNT. All three assays were in agreement determining 21 sera to be positive and 62 sera to be negative.

**Figure 2:**
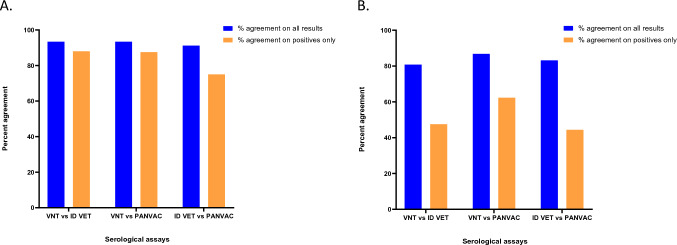
The percentage agreement of all results (blue) and positive result only (orange) when each assay is compared to another. Graph (**A**) depicts typical hosts (sheep and goat) and graph (**B**) depicts atypical hosts.

The percentage agreement for all results from typical livestock between the 3 assays was ≥ 91.2%. A Cohen’s Kappa analysis^30^, where raters include positive results and exclude negative or inconclusive results, gave kappas (k) of VNT vs ID VET ELISA = 0.83, VNT vs AU-PANVAC ELISA = 0.82 and ID VET ELISA vs AU-PANVAC ELISA = 0.77 (where a value of 1 implies perfect agreement and values less than 1 imply less than perfect agreement). When the percentage agreement for positive results only was compared, the VNT vs ID VET was 88.0%, VNT vs AU-PANVAC was 87.5% and the ID VET vs AU-PANVAC was 75.0% (Fig. 2A).

For the 702 sera from atypical hosts, the VNT detected 73 positives, 574 negatives and 55 inconclusives. The ID VET ELISA detected 109 positives, 543 negatives and 50 inconclusives and the AU-PANVAC ELISA detected 52 positives, 637 negatives and 13 inconclusive results (Fig. 1B). Although all assays detected between 52 and 109 positive sera, in many cases there was discordance between the samples determined to be positive. Of the positive results generated, 20 were unique to the VNT, 45 to the ID VET kit and two to the AU-PANVAC kit. All three assays were in agreement determining 46 sera to be positive and 491 sera to be negative. At no point in the study did all three assays determine an inconclusive result on the same serum sample.

The percentage agreement for all results from atypical hosts between the assays was ≥ 80.8% with the highest agreement seen between the VNT and AU-PANVAC ELISA, at 86.8%. The kappa was calculated as VNT vs ID VET ELISA = 0.15, VNT vs AU-PANVAC ELISA = 0.19 and ID VET ELISA vs AU-PANVAC ELISA = 0.19. The percentage agreement for positive results only showed that VNT vs ID VET was 47.5%, VNT vs AU-PANVAC was 62.3% and ID VET vs AU-PANVAC was 44.4% (Fig. 2B).

**Figure 3:**
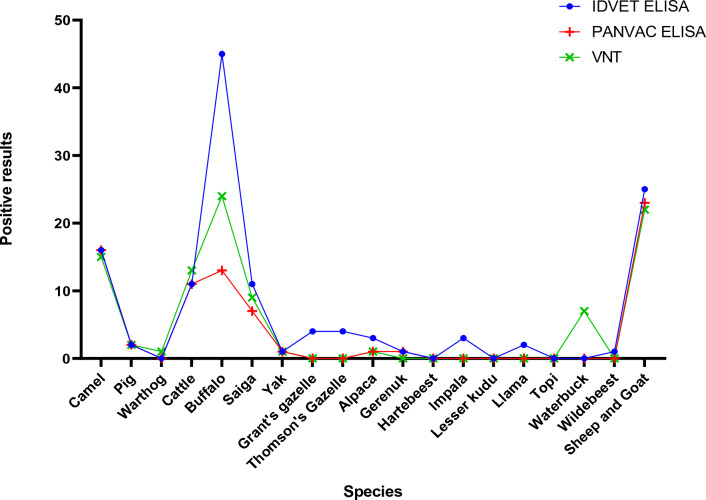
The number of positive results generated by the VNT, ID VET ELISA and AU-PANVAC ELISA testing typical and atypical species.

At the species level, positive sera of livestock sheep and goat are detected consistently between the assays but some atypical species showed much greater variation. African buffalo (n = 262) showed the greatest disagreement between assays, with the ID VET ELISA producing 45 positive results compared to the 24 and 13 determined by the VNT and the AU-PANVAC ELISA respectively. In contrast, results for dromedary (*Camelus dromedarius*, n = 105) are in far better agreement, producing 16 positive results when tested in the ID VET and AU-PANVAC ELISAs and 15 positives in the VNT (Fig. 3).

The ID VET ELISA was consistently the most sensitive of the three assays, generating the highest number of positive results across wildlife species. In some instances, such as with the Grant’s Gazelle (*Nanger granti*), Thomson’s gazelle (*Eudorcas thomsonii*), impala and llama (*Lama glama*), only the ID VET ELISA produced positive results. A notable exception were the results obtained from the waterbuck sera (n = 7) where high neutralising antibody titres identified using the VNT were not replicated in either ELISA, where no positives were found.

Of the 30 sera selected from the larger panel and sent to the Joint FAO and IAEA Centre’s Animal Production and Health Laboratory and UoG for testing using the LIPS and PVNA, respectively, there was good agreement amongst all serological assays when testing the 10 sheep and goat sera, supporting validation data for each method (Tables 2, 3). The ID VET ELISA returned positive results for all 10 typical livestock sera, one of which was unique to this assay. For the 20 atypical wildlife and domestic sera, there was far less outcome agreement. The VNT identified four African buffalo and two dromedary as inconclusive, with readable nAb titres of < 1/10. Three dromedary were deemed positive with nAbs of 1/60, 1/40 and 1/20. The ID VET ELISA identified four positive African buffalo sera, three uniquely, and also two samples from Thomson’s Gazelle and one from a Grant’s Gazelle, two of which were unique. This ELISA was in agreement with the VNT for the three positive dromedary. The AU-PANVAC ELISA was also positive for the three dromedary but identified only one positive serum from an African buffalo which was not mirrored by the ID VET kit. All other samples were negative with this assay. The LIPS was unable to detect the dromedary positives seen in the VNT and ELISAs but gave doubtful results for the four African buffalo picked up by the ID VET ELISA. Notably, it also highlighted two further positive African buffalo sera that were inconclusive in the VNT and negative in both ELISA platforms. The PVNA detected two of the three dromedary samples deemed positive by the VNT and ELISAs. It also gave positive results for four African buffalo picked up by the LIPS (n = 3) and ID VET kit (n = 1) respectively and two Thomson’s gazelle, one of which was unique to this platform. Interestingly, no single wildlife sample from this set was deemed positive by all five assays. Only four samples, comprising Grant’s gazelle (n = 2), a dromedary (n = 1) and an impala (n = 1), were deemed negative by all five assays (Tables 2, 3).

**Table 2:** A panel of 30 sera from typical and atypical species was tested using the VNT, ID VET ELISA, AU-PANVAC ELISA, LIPS and PVNA.

Host type	Sera species	VNT	ID VET cELISA	AU-PANVAC bELISA	LIPS	PVNA
Typical (livestock)	Goat	Positive	Positive	Positive	Positive	Not tested
	Goat	Positive	Positive	Positive	Positive	Positive
	Goat	Positive	Positive	Positive	Positive	Positive
	Goat	Positive	Positive	Positive	Positive	Positive
	Goat	Positive	Positive	Positive	Positive	Positive
	Goat	Positive	Positive	Positive	Positive	Positive
	Goat	Positive	Positive	Positive	Positive	Positive
	Goat	Negative	Positive	Inconclusive	Negative	Negative
	Sheep	Positive	Positive	Positive	Positive	Positive
	Sheep	Positive	Positive	Positive	Positive	Positive
Atypical (wildlife and domestic)	African buffalo	Negative	Negative	Positive	Positive	Positive
	African buffalo	Negative	Positive	Negative	Inconclusive	Negative
	African buffalo	Negative	Positive	Negative	Inconclusive	Negative
	African buffalo	Readable but < 1/10	Positive	Negative	Inconclusive	Positive
	African buffalo	Readable but < 1/10	Positive	Negative	Inconclusive	Not tested
	African buffalo	Readable but < 1/10	Negative	Negative	Positive	Positive
	African buffalo	Readable but < 1/10	Negative	Negative	Positive	Positive
	Dromedary	Positive	Positive	Positive	Negative	Positive
	Dromedary	Positive	Positive	Positive	Negative	Negative
	Dromedary	Positive	Positive	Positive	Negative	Positive
	Dromedary	Negative	Negative	Negative	Negative	Negative
	Dromedary	Readable but < 1/10	Negative	Negative	Negative	Not tested
	Dromedary	Readable but < 1/10	Negative	Negative	Negative	Negative
	Thomson’s Gazelle	Negative	Positive	Negative	Negative	Negative
	Thomson’s Gazelle	Negative	Positive	Negative	Negative	Positive
	Thomson’s Gazelle	Negative	Negative	Negative	Negative	Positive
	Grant’s Gazelle	Negative	Positive	Negative	Negative	Negative
	Grant’s Gazelle	Negative	Negative	Negative	Negative	Negative
	Grant’s Gazelle	Negative	Negative	Negative	Negative	Negative
	Impala	Negative	Negative	Negative	Negative	Negative

The positive, negative and inconclusive (readable but < 1/10 for VNT) results are displayed against each species.

**Table 3:** For the 30 sera panel tested, the total positive, unique positive and inconclusive (< 1/10 for VNT) results are displayed.

Host type	Result	Assay				
		VNT	ID VET cELISA	AU-PANVAC bELISA	LIPS	PVNA^c^
Typical^a^ n = 10	Total positive	9	10	9	9	8
	Unique positive	0	1	0	0	0
	Inconclusive (< 1/10 VNT)	0	0	1	0	0
Atypical^b^ n = 20	Total positive	3	10	4	3	8
	Unique positive	0	5	0	0	1
	Inconclusive (< 1/10 VNT)	6	0	0	4	0

^a^Typical species comprised eight goats and two sheep.

^b^Atypical species comprised six dromedary, seven African buffalo, three Grant’s gazelle, three Thomson’s gazelle and one impala.

^c^Three sera (one goat, one African buffalo and one dromedary) were not tested by PVNA following a logistical issue.

## Discussion

In order to protect biodiversity and support a PPRV vaccination campaign in domestic goats and sheep it is crucial that there is credible evidence that free-ranging wildlife populations, sharing the same geographical locations with goat and sheep populations through complex interfaces, do not have the capacity to harbour and reintroduce the virus to domestic stock at a later date. Although the dynamics of this wildlife-livestock interface are the subject of other recent publications^2,6,7,10,31,32^, suggesting low seroprevalence with additional molecular evidence of PPRV circulation, these rely on having confidence in the surveillance and testing methodologies employed. This is problematic when the frontline serological assays for such a purpose have been optimised and validated using typical host sera only, largely omitting wild and domestic atypical species. Indeed, the application of the term ‘atypical’ is contentious as this is often used to describe wildlife as a whole and does not account for the many wildlife species of the Capra (goat) and Ovis (sheep) genuses that may contract, suffer from and spread PPRV in the same manner as livestock sheep and goats. Also, some members of the sub-family Antilopinae, such as saiga, have been observed to produce a severe clinical response to infection and could transmit the disease, at least at the intra-species level^12^. This is not the case in larger free-ranging bovids, such as African buffalo, which are considered dead-end hosts outside of captive settings^8,32^. It may, therefore, be more prudent to think of atypical host species as those being either epidemiologically significant or not epidemiologically significant as this is more conducive to forming adequate testing and vaccination campaigns. Many wild species may be thought of as being epidemiologically insignificant or dead-end hosts due to their lack, or variation, of a traditional presentation of the disease (pyrexia, nasal congestion, diarrhoea and bronchopneumonia) and little or no data demonstrating transmission to other species^8^. However, the importance of dead-end hosts as sentinel animals for tracking virus circulation in endemic regions is substantial^14,33^. Atypical species may respond differently to disease under conditions of captivity and related stress. There is limited experimental evidence that species of the Suidae family may play a role in transmission and act as reservoir hosts, thus the ID VET cELISA kit was recently validated for use with pig sera^9,17^.

The VNT is a useful tool for demonstrating evidence of previous infection and/or vaccination by detecting neutralising antibodies in the serum, however viable virus (attenuated or field, capable of producing CPE, syncytia or carrying a reporter gene such as GFP) is needed, necessitating the need for a containment laboratory to mitigate the risk of virus release. Such laboratories are costly to build and maintain, requiring specialist infrastructure and engineering. The ELISA and LIPS do not require the use of viable virus and the PVNA uses a replication defective pseudotyped virus which is incapable of causing disease, therefore negating the need for containment laboratories. Unlike the VNT, the ELISAs, LIPS and PVNA are target-specific, revealing antibodies against specific proteins or peptide antigens. The LIPS and PVNA can be easily customised by changing the sequences in the constructs used to produce the fusion protein and pseudotyped virus surface glycoproteins respectively, enabling the user to target different conserved regions between lineages and other regions altogether. This is beneficial when serological cross-reactivity between morbilliviruses can obscure the true neutralising antibody titre against PPRV and cast doubt over results^19^. The *Morbillivirus* genus also includes measles virus (MV), canine distemper virus (CDV) and the eradicated rinderpest virus (RPV). These closely related viruses have been demonstrated to show serological cross reactivity to PPRV^19,22^ which can hamper traditional methods of antibody detection.

Both the VNT and PVNA are cell-culture based assays and therefore the facilities and expertise to culture susceptible mammalian cell lines are required. Morbilliviruses such as PPRV and pseudotyped viruses require the signalling lymphocyte activating molecule (SLAM) receptor to facilitate cellular entry and further propagation, hence Vero or HEK293-derived target cells, stably expressing the goat or canine SLAM receptors, have been developed and are widely available^19,34^. Similar genetic manipulation is also required to produce the fusion protein used in the LIPS and the pseudotyped viruses used in the PVNA^1,19^.

The suitability of each serological assay is dependent on the disease status of a given region, the sensitivity and specificity of the assay and the availability of the necessary infrastructure required to perform them safely and successfully. The data presented here demonstrate the importance of considering the target species when choosing an assay. The results of this study show that the gold standard VNT and two commercially available ELISA kits can be relied upon to provide sensitive and consistent serological data for PPRV when used to test sera from domestic sheep and goats (and potentially closely related free-ranging relatives). There is also promising data that the LIPS and PVNA under development may be in line with this, though a greater sample size will be needed for statistical confidence. The same cannot be applied to sera from atypical species, in their entirety, as there is evident inter-species disagreement between the five assays tested. The largest sera sets from atypical species tested were the African buffalo (n = 262), dromedary (n = 105) and Grant’s gazelle (n = 147). For African buffalo, the ID VET ELISA returned nearly double the number of positive results when compared to the VNT and more than three times that of the AU-PANVAC ELISA. Of the 45 ID VET positives, 27 of these were unique although eight of these sera had readable nAb titres < 1/10 in the VNT. There were only eight cases of sera being identified as positive by the VNT and/or AU-PANVAC that were not detected by the ID VET kit. Although African buffalo play an insignificant role in PPRV transmission their epidemiological relevance when used to assess the level of virus circulation in vaccinated areas is considerable, thus highlighting the importance of reliable serological tests for bovids^16^. Our data could also indicate circulation of antigenically-related morbilliviruses that may confound reliable diagnosis in this species, meriting further investigation. By comparison, agreement over the 16 positive dromedary sera identified was nearly 100% between the three tests, contrasting a previously published study^35^. Several additional nAb titres < 1/10 were detected in the dromedary sera by the VNT when a low, initial dilution of sample was tested.

There were many examples where, for some sera, the ID VET ELISA produced a positive result that the other serological assays did not corroborate. An example was the Grant’s gazelle sera, where the four positives and seven inconclusive results were unique to the ID VET kit. Similar cases were seen in Thomson’s gazelle (four positive, one inconclusive), impala (three positive, four inconclusive) and llama (two positive). Though this may be seen as evidence of high sensitivity, the low agreement with the VNT gold standard may raise questions over specificity. The evaluation of specificity can be further addressed in later studies by testing known-negative samples from areas free of PPR, for example South Africa. It is, perhaps, predictable that the VNT shares a higher agreement with the AU-PANVAC ELISA across the majority of the samples from atypical species as both assays target neutralising antibodies (anti-H and anti-F (VNT) antibodies). An exception to this was seen with the six waterbuck sera which, when tested using the VNT, neutralised rPPRV/eGFP Nig 75/1 at dilutions beyond 1/3840 in some cases (data not shown). Despite this, no N or H antibodies were detected in these sera using the ELISAs, suggesting either action by anti-F antibodies or another unknown blood chemistry causing non-specific reactions. This phenomenon was also identified amongst waterbuck species during serological testing for Rinderpest^36^. The quality and/or heterogeneity of the samples used, as well as possible circulation of other morbilliviruses, may account for some of the variation observed amongst the assays.

The results of the LIPS and PVNA methods in atypical species were varied. The LIPS identified only African buffalo sera as being positive or inconclusive, with all other atypical sera deemed negative. Two of the African buffalo sera positive in the LIPS, however, were not detected by either ELISA. These were mirrored by the PVNA, which largely agreed with the VNT on African buffalo and dromedary samples, perhaps unsurprisingly given the similarities in methodology. It also detected two Thomson’s gazelle positives not seen in the VNT, AU-PANVAC ELISA or LIPS. Expanding the data set to include further examples of different species tested by the LIPS and PVNA is necessary to gain a statistically confident evaluation of their use as serological tests for wildlife and domestic atypical hosts.

## Conclusion

The serological assays presented here demonstrate high sensitivity, specificity and agreement when used for antibody detection in sera from domestic sheep and goats, considered typical hosts and epidemiologically significant. Wildlife and other domestic species should be separated into epidemiologically significant or insignificant categories rather than being considered ‘atypical’ in all cases. Our data suggest that the sensitivity and specificity of currently available serological tests is dependent upon which species is being tested, with wildlife species more closely related to ovine and caprine species being more likely to produce consistent results where multiple serological assays are used. The ID VET ELISA appeared to show the greatest sensitivity among the five assays and is arguably the simplest test to deploy in areas with limited resources, however positive results from this test were often not corroborated by the AU-PANVAC ELISA or VNT for atypical species. This may indicate that the latter assays lack sensitivity, or that the enhanced sensitivity of the ID VET ELISA renders it more likely to detect responses elicited by atypical infections with antigenically-related species of morbillivirus such as CDV. The LIPS and PVNA may provide a greater specificity and limit issues with serological cross-reactivity due to their customisation of target proteins. The testing of a greater variety of atypical sera (including from PPR-free areas) using these tests, and a larger sample size of species thought to be significant in the global eradication of PPRV, are recommended future endeavours. This will promote the development of a protocol for wildlife PPR testing, according to species sensitivity, supporting sero-surveillance efforts and vaccination campaigns.

### Supplementary Information

Supplementary Tables.

## Data Availability

The corresponding author can provide the datasets for this work upon reasonable request.
