# Supplementary material for: The evaluation of five serological assays in determining seroconversion to peste des petits ruminants virus in typical and atypical hosts
Source: Sci Rep. 2023 Sep 8;13:14787. doi: 10.1038/s41598-023-41630-3 (PMC10491793; doi:10.1038/s41598-023-41630-3)
Supplement: Supplementary file 1 — Supplementary Tables. [file 41598_2023_41630_MOESM1_ESM.docx]

**Supplementary data**

| **Virus Neutralisation Test (VNT)** | |
| --- | --- |
| Neutralising antibody (nAb) titre of 1/10 or above | Positive |
| nAb titre below test threshold | Negative |
| Apparent contamination or resulting cytotoxicity from a source other than virus OR nAb titre above test threshold but <1/10 | Inconclusive |
| **ID VET cELISA** | |
| Competition percentage (S/N%) >60% | Negative |
| 50% < S/N% <= 60% | Doubtful |
| S/N% <= 50% | Positive |
| **ID VET cELISA (Suidae species only)** | |
| S/N% >40% | Negative |
| 30% < S/N% <= 40% | Doubtful |
| S/N% <= 30% | Positive |
| **AU-PANVAC bELISA** | |
| Percentage Inhibition (PI) <= 30% | Negative |
| 30% < PI < 35% | Doubtful |
| PI >= 35% | Positive |
| **Luciferase Immunoprecipitation System (LIPS)** | |
| Relative Light Units (RLU) < Mean negative RLU + 3 Standard Deviations (Std) | Negative |
| RLU > Mean negative RLU + 5 Std | Positive |
| Mean negative RLU +3 Std < RLU < Mean negative RLU +5 Std | Borderline |
| *Note: The Mean negative RLU for Wildlife (Atypical) and Livestock (Typical) species were ascertained previously from known PPRV Ab negative sera (data not shown)* | |
| **Pseudovirus Neutralisation Assay (PVNA)** | |
| Neutralising antibody (nAb) titre derived above test threshold | Positive |
| nAb titre below threshold | Negative |

**Table S1:** Results ranges and criteria for each assay performed in this study

|  |  |  | Assay results | | | | |
| --- | --- | --- | --- | --- | --- | --- | --- |
| Country of origin | Species | Host type | VNT nAb titre | ID VET cELISA S/N% | AU-PANVAC bELISA PI | LIPS RLU | PVNA nAb titre |
| Sudan  Sudan  Sudan  Sudan  Sudan  Sudan | Dromedary | Atypical | 1/60 | 21.33 | 65.34 | 276.50 | 55.60 |
|  | Dromedary |  | 1/40 | 27.73 | 63.28 | 216.00 | <32 |
|  | Dromedary |  | 1/20 | 27.40 | 35.58 | 263.00 | 294.86 |
|  | Dromedary |  | 1/3.75 | 67.02 | 28.28 | 215.50 | Not tested |
|  | Dromedary |  | 1/3.75 | 74.49 | 18.26 | 192.00 | <32 |
|  | Dromedary |  | <1/10 | 116.08 | 17.22 | 263.00 | <32 |
| Tanzania | African buffalo |  | <1/2.5 | 60.01 | 35.06 | 1655.50 | 49.47 |
| Tanzania | African buffalo |  | <1/2.5 | 39.41 | 18.61 | 579.00 | <32 |
| Tanzania | African buffalo |  | <1/2.5 | 45.78 | 18.19 | 539.00 | <32 |
| Tanzania | African buffalo |  | 1/2.5 | 30.96 | 20.67 | 610.50 | 80.68 |
| Tanzania | African buffalo |  | 1/5 | 49.77 | 26.52 | 681.50 | Not tested |
| Tanzania | African buffalo |  | 1/5 | 78.57 | 21.72 | 1489.50 | 89.33 |
| Tanzania | African buffalo |  | 1/3.75 | 69.50 | 24.57 | 1118.50 | 34.52 |
| Tanzania | Grant’s Gazelle |  | <1/2.5 | 43.30 | -25.53 | 142.00 | <32 |
| Tanzania | Grant’s Gazelle |  | <1/10 | 100.83 | -4.71 | 268.00 | <32 |
| Tanzania | Grant’s Gazelle |  | <1/10 | 106.07 | -17.91 | 121.00 | <32 |
| Tanzania | Thomson’s Gazelle |  | <1/2.5 | 24.77 | 0.37 | 101.25 | <32 |
| Tanzania | Thomson’s Gazelle |  | <1/2.5 | 26.03 | 21.58 | 130.25 | 32.71 |
| Tanzania | Thomson’s Gazelle |  | <1/10 | 87.99 | 2.04 | 356.75 | 131.11 |
| Tanzania | Impala |  | <1/10 | 63.67 | 21.72 | 71.00 | <32 |
| Tanzania | Goat | Typical | 1/160 | 4.15 | 71.28 | 101157.50 | Not tested |
| Tanzania | Goat |  | 1/320 | 3.20 | 87.24 | 153238.00 | 75.85 |
| Tanzania | Goat |  | 1/160 | 4.67 | 81.72 | 80402.00 | 75.86 |
| Tanzania | Goat |  | 1/320 | 6.32 | 92.46 | 15932.75 | 106.48 |
| Tanzania | Goat |  | 1/80 | 0.52 | 84.88 | 46147.25 | 105.18 |
| Tanzania | Goat |  | 1/320 | 0.95 | 84.09 | 52881.25 | 103.14 |
| Tanzania | Goat |  | 1/320 | 4.07 | 94.93 | 170889.25 | 81.21 |
| Tanzania | Goat |  | <1/2.5 | 44.14 | 30.49 | 131.50 | <32 |
| Tanzania | Sheep |  | 1/120 | 3.72 | 85.76 | 4091.75 | 80.43 |
| Tanzania | Sheep |  | 1/120 | 4.50 | 88.33 | 25945.75 | 97.06 |

**Table S2:** Raw results data of the n = 30 panel tested using the VNT, ID VET ELISA, AU-PANVAC ELISA, LIPS and PVNA.
